# Effect of *Helicobacter pylori* eradication on ongoing mutation of immunoglobulin genes in gastric MALT lymphoma

**DOI:** 10.1038/sj.bjc.6602262

**Published:** 2005-01-11

**Authors:** K Fujimori, S Shimodaira, T Akamatsu, K Furihata, T Katsuyama, S Hosaka

**Affiliations:** 1 The Second Department of Internal Medicine, Shinshu University School of Medicine, Matsumoto, Japan; 2 Department of Endoscopy, Shinshu University School of Medicine, Matsumoto, Japan; 3 Department of Laboratory Medicine, Shinshu University School of Medicine, Matsumoto, Japan; 4 Department of Internal Medicine, Maruko General Hospital, 335-5 Maruko-machi, Chisagata-gun, Nagano-ken 386-0493, Japan

**Keywords:** gastric MALT lymphoma, immunoglobulin heavy chain gene, ongoing mutation, *Helicobacter pylori* eradication

## Abstract

Gastric low-grade mucosa-associated lymphoid tissue (low-grade MALT) lymphomas has been associated with *Helicobacter pylori* (*H. pylori*) infection. Although infiltrating T cells with specificity for *H. pylori* are known to stimulate the development of MALT lymphomas, the effect of *H. pylori* eradication on rearranged immunoglobulin heavy chain (IgH) genes of low-grade gastric MALT lymphomas is unclear. Gastric biopsies from five cases were investigated by cloning and sequence analysis of rearranged IgH genes before and after the treatment for *H. pylori*. In all cases, IgH genes were mutated from their germline counterpart. The frequency of intraclonal sequence heterogeneity before the eradication of *H. pylori* varied from 0.25 to 0.49%. Clones obtained from the tumours before the eradication of *H. pylori* in cases 1 and 2 showed a tendency to display a mutation pattern by positive antigen selection and their monoclonarity disappeared after the eradication. The frequency of intraclonal sequence heterogeneity of the clones obtained from cases 3, 4 and 5 (0.12% in case 3, 0.10% in 4 and 0.18% in 5) after the eradication of *H. pylori* was lower than that in tumours before the eradication (0.30% in case 3, 0.49% in 4 and not determined in 5). These findings suggest that low-grade gastric MALT lymphomas expand due to the persistent presence of *H. pylori in vivo*. The characteristic feature of tumour clones obtained from the tumours after the eradication of *H. pylori* is a very low intraclonal heterogeneity, which may potentially be independent of *H. pylori*.

In response to antigen stimulation, the reaction of the germinal centre of lymphoid follicles generates the memory B cells, producing antibodies with a high affinity and specificity (Maclennan and Gray, 1989; Hentges, 1994). Concerning the somatic hypermutations in the rearranged immunoglobulins heavy chain (IgH) variables (V_H_) gene of the memory B cells, the ration of replacement (R) to silent (S) mutations in the complementary determining regions (CDRs) was observed to be higher than that in the frame work regions (FRs) (Both *et al*, 1990; Jacob *et al*, 1991). Sequence analysis of rearranged V_H_ gene provides information about the mutation stage of B-cell differentiation and can help assess the role of antigen stimulation in clonal selection and expansion (Stewart and Schwartz, 1994). Tumour cells derived from germinal- to postgerminal-centre B cells such as those of follicular lymphoma and multiple myeloma have shown extensive somatic mutations (Bahler and Levy, 1992; Bakkus *et al*, 1992; Zelenetz *et al*, 1992; Sahota *et al*, 1996). In contrast, tumour cells from chronic lymphocytic leukaemia and mantle cell lymphoma have been generally unmutated and are thought to be derived from pregerminal-centre B cells (Meeker *et al*, 1988; Zhu *et al*, 1994).

Ongoing mutation, which indicates an intraclonal sequence heterogeneity, shows that lymphoma cells continuously react to antigens in lymphomagenesis (Bakkus *et al*, 1992; Zelenetz *et al*, 1992). In mucosa-associated lymphoid tissue (MALT) lymphoma, frequent and ongoing mutations in the rearranged V_H_ gene have been reported, providing the evidence of positive antigen selection during the germinal-centre reaction (Qin *et al*, 1995; Du *et al*, 1996). Infection of *Helicobacter pylori* (*H. pylori*) plays an important role in the development of gastric MALT lymphoma by acting as an indirect antigen stimulation for the *H. pylori*-specific T cell implicated in the development of tumours (Hussell *et al*, 1993); however, the role of *H. pylori* eradication in ongoing mutations in the immunoglobulin V_H_ gene is still unclear.

In this study, we cloned and sequenced CDRs and FRs of rearranged V_H_ genes before and after the eradication of *H. pylori* in gastric MALT lymphomas to determine the role of *H. pylori* infection in ongoing mutation in lymphomagenesis.

## MATERIALS AND METHODS

### Patients

Five cases of primary gastric MALT lymphoma that had undergone treatment for *H. pylori* were investigated (Table 1). For the endoscopic examinations before and after treatment, three to 10 specimens were taken from tumours or suspicious areas for histology, and two to five others were taken for PCR. The diagnosis of MALT lymphoma was based on the criteria described by Isaacson (Isaacson and Norton, 1994) and the WHO classification (Harris *et al*, 1999) by staining with haematoxylin and eosin and immunohistochemical examination on paraffin-embedded tissues from gastric biopsy specimens. The histological findings from the gastric MALT lymphoma were graded with the histologic scoring system (Wotherspoon *et al*, 1993). The surface phenotypes were analysed by flow cytometry in cases 1, 2 and 5 (Table 1). The *H. pylori* status was assessed in eight biopsy specimens as described previously (Shimizu *et al*, 1996), and was detected in all cases. Patients were treated for *H. pylori* infection for 2 weeks with lansoprazole (30 mg day^−1^) and amoxicillin (1500 mg day^−1^), or lansoprazole (30 mg day^−1^), metronidazole (750 mg day^−1^), and amoxicillin (1500 mg day^−1^) or clarithromycin only (600 mg day^−1^) (Shimizu *et al*, 1996). At 6 weeks after treatment (4 weeks after completion of treatment), the eradication of *H. pylori* and MALT lymphoma status was endoscopically assessed. Thereafter, patients were followed up every 2–6 months. It was found that the complete eradication of *H. pylori* had been achieved in all cases.

The patients' clinical and follow-up data obtained before and after the treatment for *H. pylori* are summarised in Table 1.

Cases 1 and 2 had low-grade gastric MALT lymphomas and were treated for *H. pylori*, and the eradication of *H. pylori* was accompanied by improvement in both the endoscopic and histological grades.

Case 3 had a low-grade MALT lymphoma with multiple ulceration in the greater curvature of the gastric corpus. At 9 months after treatment for *H. pylori*, the endoscopic and histological findings had not improved. Next, the patient was given 50 mg day^−1^ of cyclophosphamide for 7 months, again with no improvement in the lymphoma. When *H. pylori* was detected again, the patient received a second treatment to eradicate the *H. pylori*. The treatment was successful and subsequent analysis showed that the endoscopic findings had improved, but not the histological grade.

In case 4, diagnosed as low-grade MALT lymphoma, *H. pylori* was successfully eradicated but no endoscopic or histological regression was seen, and 6 months later, the sigmoid colon has also become involved. Sequence analysis was therefore performed again at this time.

Case 5 had a 7-year history of recurrent gastric ulcer, and low-grade MALT lymphoma accompanied by *H. pylori* infection was histologically identified. At 28 months after treatment for *H. pylori*, the patient was diagnosed as having high-grade components in the same area as the recurrent ulcer but without *H. pylori* and diagnosed with diffuse large B-cell lymphoma (DLBCL). The clinical stage was stage I, and the patient underwent a total gastrectomy and splenectomy.

### Extraction of DNA from tumour tissue

Gastric biopsy specimens from the tumour areas before treatment for *H. pylori* and the areas with residual tumours after the eradication of *H. pylori* (available only after treatment for *H. pylori* in case 5) were used as a source of tumour DNA for the analysis of the IgH gene sequence. The Raji B-cell line was used as a monoclonal control, and normal peripheral blood was used as a polyclonal control. Genomic DNA was extracted from fresh tissues using the modified phenol–chloroform extraction procedure described previously (Sambrook *et al*, 1989).

### Clonal analysis of rearranged IgH gene by PCR

A PCR was performed in a 1605 Air Thermo-Cycler (Idaho Technology Inc., Idaho Falls, ID, USA). Genomic DNA was amplified in a final volume of 25 *μ*l reaction buffer (50 mM Tris-HCl (pH 8.3), 1.5 mM MgCl_2_, 250 *μ*g ml^−1^ BSA) containing 0.75 U of Taq DNA polymerase (Perkin-Elmer, Norwalk, CT, USA), 200 *μ*M of dNTPs (Idaho Technology), 1 *μ*M of an upstream consensus primer specific for the FR2 of the IgH variable region (V) gene segments (FR2B: 5′-GTCCTGCAGGC[C/T][C/T]CCGG[A/G]AA[A/G][A/G]GTCTGGAGTGG-3′) and a downstream primer specific for joining region (J) gene segments (ELJH: 5′-TGAGGAGACGGTGACCAGGATCCCTTGGCCCAG-3′) (Ramasamy *et al*, 1992). Following an initial denaturation step at 94°C for 1 min and an initial two cycles of amplification (denaturation at 98°C for 15 s, annealing at 60°C for 20 s, extension step at 72°C for 30 s), 43 cycles of amplification were performed (denaturation at 98°C for 15 s, annealing at 60°C for 15 s, extension step at 72°C for 30 s), followed by a final extension step at 72°C for 2 min. If the reaction could not be amplified, a seminested PCR was used. For the first PCR round, amplification was carried out under the same conditions as described above, except that 28 cycles were performed instead of 43, with an upstream consensus primer specific for the FR2 region of V gene segments (FR2A: 5′-TGG[A/G]TCCG[C/A]CAG[G/C]C[T/C][T/C]CNGG-3′) (Sambrook *et al*, 1989) or the FR3 region (FR3A: 5′-ACACGGC[C/T][G/C]TGTATTACTGT-3′) (Brisco *et al*, 1990; Ramasamy *et al*, 1992) and a downstream primer specific for J gene segments (LJH: 5′-TGAGGAGACGGTGACC-3′) (Brisco *et al*, 1990; Ramasamy *et al*, 1992). For the second round, 1 *μ*l of the first-round products was amplified under the same conditions as described above, except that 18 cycles were performed instead of 43, with FR2A or FR3A and VLJH (5′-GTGACCAGGGTNCCTTGGCCCCAG-3′) (Brisco *et al*, 1990; Trainor *et al*, 1990). To confirm the monoclonal B-cell proliferation, aliquots of the final reaction products were subjected to 6% (FR2B-ELJH and FR2A-VLJH products) or 10% (FR3A-VLJH products) polyacrylamide gel electrophoresis and were subsequently stained with ethidium bromide.

### Cloning and sequencing of IgH genes

The PCR products were electrophoresed through a 3% agarose gel and were purified with Geneclean II (Bio 101 Inc., Vista, CA, USA). The purified products were ligated into a pCR™II vector and then used to transform INV*α*F′-competent cells according to the manufacturer's protocol (Invitrogen, Leek, The Netherlands). Nine to 12 white colonies were picked at random and were grown overnight in 2 ml of Luria–Bertani medium. When clones were found to contain an insert of an appropriate size by a restriction analysis of the plasmid DNA, they were sequenced by the dideoxy chain termination method using the ABI model 373A DNA sequencer (Applied Biosystems Inc., Foster, CA, USA) with an M13 forward primer and an M13 reverse primer.

### Sequence analysis

A predominant V sequence with an identical CDR3 was assumed to be a consensus tumour-derived clone. A sequence alignment analysis was carried out to compare with current GenBank and V-BASE sequence directories (MRC Centre for Protein Engineering, Cambridge, UK) (Cook and Tomlinson, 1995) using MacVector 4.0 sequence analysis software (International Biotechnologies Inc., New Haven, CT, USA). The closest germline V genes and their degree of similarity to each consensus tumour-derived clone were determined. Mutations in the variable regions were identified by comparing the consensus sequence of each clone with the closest published germ line. Two nucleotide exchanges in a single codon were scored as one replacement mutation. Diversity (D) and J regions were also determined by their corresponding published germ line D and J sequences, respectively.

### Somatic mutation analysis

To detect positive antigen stimulation, the number of expected somatic mutations in the rearranged V sequences was calculated before eradication (cases 1–4) and after eradication (case 5) as follows. For *n* random mutations, the number of expected replacement (R) mutations should equal 0.75*n*, and the number of expected silent (S) mutations should equal 0.25*n*. Without selection, the R and S mutations should be distributed among the various V regions according to their respective sizes, that is, RCDRs=CDRrel × R, RFRS=FRrel × R, SCDRs=CDRrel × S and SFRs=FRrel × S (CDRrel and FRrel, relative size of CDRs or FRs) (Bahler and Levy, 1992; Du *et al*, 1996). The probability (*P*) that the observed R mutations in the CDRs were chance occurrences was calculated by using a binomial mutation model: *P*=[*n*!/*k*!(*n*−*k*!)!]*q*^*k*^(1−*q*)^*n*–*k*^, where *k* is the number of observed R mutations in the CDRs and *q* is the probability that an R mutation will localise to CDRs (Zelenetz *et al*, 1992; Chang and Casali, 1994). Intraclonal sequence heterogeneity was calculated according to the method of Du *et al*.

## RESULTS

### Cloning analysis of rearranged IgH genes by PCR

All four cases (1–4) of low-grade gastric MALT lymphomas were shown to have a monoclonal pattern before treatment for *H. pylori*. After the eradication of *H. pylori*, identical monoclonal bands disappeared in cases 1 and 2 but remained in cases 3–5 as shown in Table 1.

### V gene usage

**Figure 1:**
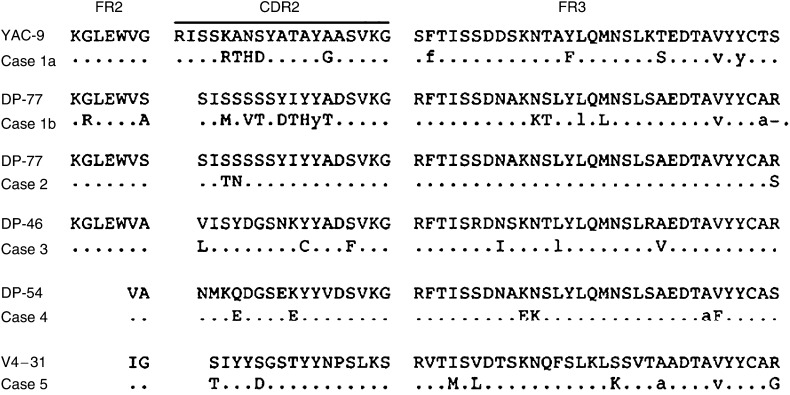
Deduced amino-acid sequence of the V regions. Comparisons are made with the closest germline genes. Upper case letters, replacement mutations; lower case letters, silent mutations. The sequences of the PCR primers of FR2A or FR2B have been excluded.

Fresh specimens obtained from cases 1 to 4 before the eradication of *H. pylori* were available for V_H_ gene analysis. Sequence analysis of the IgH gene was also carried out after the eradication of *H. pylori* in cases 3–5. Case 1 had two predominant sequences, suggesting the presence of biclonal tumour-derived clones (cases 1a and b), while cases 4 and 5 showed a single predominant V sequence with an identical CDR3 sequence. A predominant sequence from case 2 was identified with only two of 11 clones. In this case, there appeared to be a minor population of tumour cells in the biopsy specimens at the posterior wall of the stomach. In case 3, a tumour-derived sequence was identified with eight of nine clones. The V genes used by the predominant clones and the percentage of homology to their closest germline counterpart are shown in Table 2. The deduced amino-acid sequences of the predominant clones, as compared with their germline counterpart, are shown in Figure 1. It was demonstrated that four of five clones were derived from the V3 family and the remaining one from the V4 family. The percentage of homology with the germline donor in cases 1, 3, 4 and 5 varied from 88.2 to 96.4%, whereas in case 2, the rearranged sequence showed a high degree of homology (98.2%) (Table 2). No preferential germline donor of V genes was detected in any of the five cases.

### D and J genes segments

Analysis of the J genes showed that all five cases used a J4 gene segment. No preferential usage of the D segment was observed, while the probable germline D segments used are shown in Table 2.

### Somatic mutation analysis

The number of R mutations in the CDR and FR regions is shown in Tables 2 and 3. When nucleotide substitutions were attributable to somatic mutations, these cases had a much higher R-to-S mutation ratio in the CDRs than the FRs. In all cases, the Ig genes had mutated from their germline counterpart. The number of expected R and S mutations was calculated by assuming, in the absence of selection, that the distribution of R and S mutations among the various V regions would be in accordance with their respective sizes. In cases 1–3, the number of R mutations seen in the CDRs was larger than that could be expected for chance occurrence. In contrast, there was no significant concentration of R mutations in the CDRs in cases 4 and 5.

### Analysis of intraclonal sequence heterogeneity

**Figure 2:**
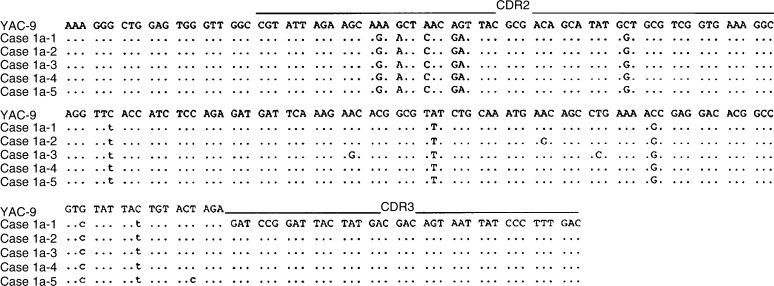
Intraclonal heterogeneity of the nucleotide sequences of the V genes used by one of two predominant tumour clones from case 1. Case1a-1 through case1a-5 represents individual cloned sequences that are compared with the closest gene, YAC-9. Silent or replacement mutations are indicated below the sequences, in lower case or upper case letters, respectively. The sequences of the PCR primers (both FR2A and VLJH) have been excluded.

**Figure 3:**
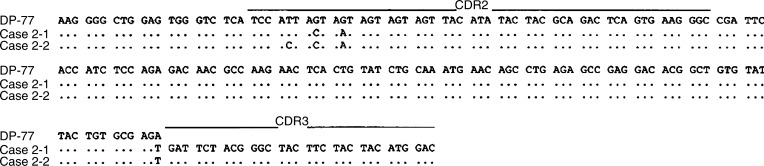
Intraclonal heterogeneity of the nucleotide sequences of the V genes from case 2. Case 2-1 through case 2-2 represents individual cloned sequences that are compared with the closest gene, DP-77. Silent or replacement mutations are indicated below the sequences, in lower case or upper case letters, respectively. The sequences of the PCR primers (both FR2A and VLJH) have been excluded.

**Figure 4:**
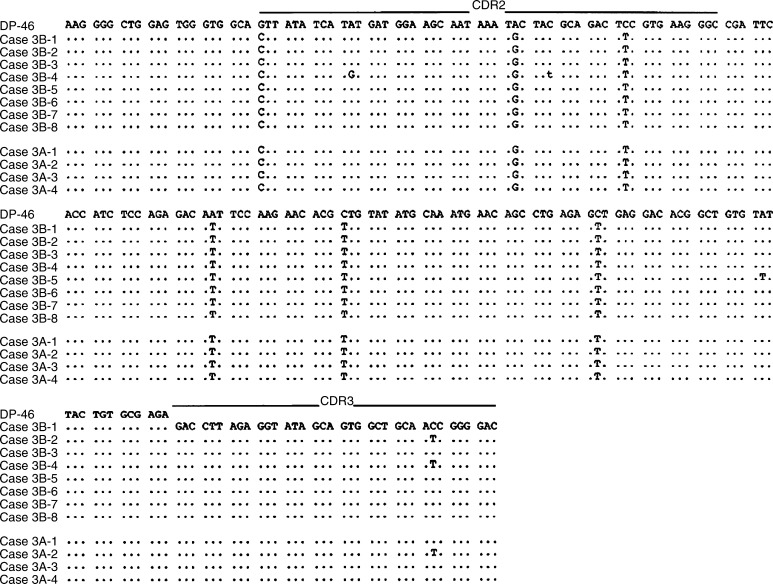
Intraclonal heterogeneity of the nucleotide sequences of the V genes from case 3 before and after the eradication of *H. pylori*. Case 3B: before eradication; case 3A: after eradication. Case 3B-1 through case 3A-8 represents individual cloned sequences that are compared with the closest gene, DP-46. Silent or replacement mutations are indicated below the sequences, in lower case or upper case letters, respectively. The sequences of the PCR primers (both FR2A and VLJH) have been excluded.

**Figure 5:**
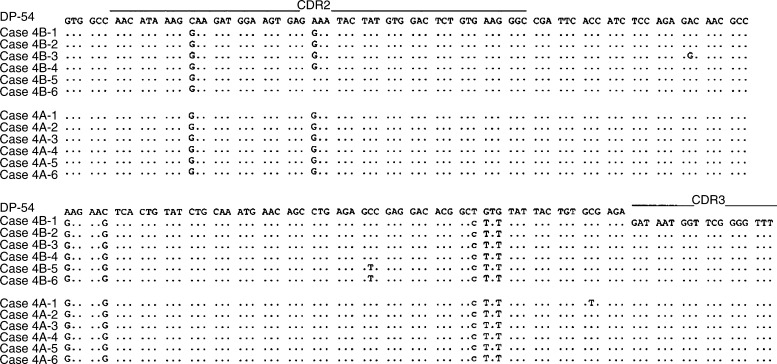
Intraclonal heterogeneity of the nucleotide sequences of the V genes from case 4 before and after the eradication of *H. pylori*. Case 4B: before eradication; case 4A: after eradication. Case 4B-1 through case 4A-6 represents individual cloned sequences that are compared with the closest gene, DP-54. Silent or replacement mutations are indicated below the sequences, in lower case or upper case letters, respectively. The sequences of the PCR primers (both FR2B and ELJH) have been excluded.

**Figure 6:**
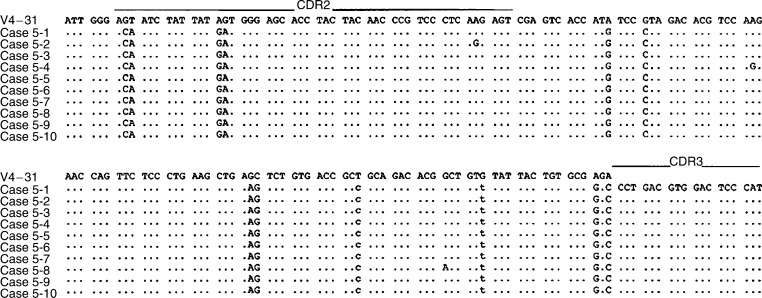
Intraclonal heterogeneity of the nucleotide sequences of the V genes from case 5. Case 5-1 through case 5-10 represents individual cloned sequences that are compared with the closest gene, V4–31. Silent or replacement mutations are indicated below the sequences, in lower case or upper case letters, respectively. The sequences of the PCR primers (both FR2B and ELJH) have been excluded.

In all of the cases studied, the sequence heterogeneity among the tumour-derived clones in each case was observed as shown in Table 4 and Figures 2, 3, 4, 5 and 6. The frequency of the sequence heterogeneity in an identical clone varied from 0.25 to 0.49% before eradication of *H. pylori* and was found to be 0.18% in case 5, with none showing any ongoing mutations as shown in Figure 6. Although two predominant sequences were detected in case 1 before eradication, only case 1a showed intraclonal heterogeneity as shown in Figure 2. Of the only two tumour-derived sequences that could be obtained from case 2 before eradication, intraclonal heterogeneity was observed as shown in Figure 3 and Table 4.

### Comparative analysis of B-cell clones before and after the eradication of *H. pylori*

Changes in the intraclonal sequence heterogeneity were also recognised after treatment for *H. pylori* (Figures 4, 5 and 6). In cases 3 and 4, intraclonal heterogeneity was observed before and after the eradication of *H. pylori*. Before the eradication of *H. pylori*, in case 3, a total of five mutations were shown in four of eight clones, and one mutation was shared in two of eight clones as shown in Figure 4. The sequence heterogeneity was found in three of six clones from case 4, and two of six clones shared two mutations as shown in Figure 5. However, after the eradication of *H. pylori*, cases 3 and 4 demonstrated only one mutation in one tumour-derived sequence, and the frequencies of the sequence heterogeneity decreased to 0.10 and 0.12%, respectively, indicating that there was no intraclonal heterogeneity, similar to the finding for the DLBCL (case 5) as shown in Figure 6.

## DISCUSSION

Analyses of the Ig gene in low-grade gastric MALT lymphoma have demonstrated the presence of somatic hypermutation (Qin *et al*, 1995) and intraclonal heterogeneity (Du *et al*, 1996). In the study presented here, ongoing mutations of rearranged IgH genes were observed in four gastric MALT lymphomas before the eradication of *H. pylori*. These results suggest that gastric low-grade MALT lymphomas derive from hypermutated postgerminal-centre memory B cells, which have undergone antigen selection as described by Qin *et al* and Du *et al*. In our study, intraclonal sequence heterogeneities of the IgH genes remained even after the eradication in three cases with lower frequencies than before the eradication of *H. pylori*. These findings suggest that there may be an ongoing mutation independent of *H. pylori* infection or clonal selection in the pathogenesis of lymphoma. Comparison of intraclonal sequence heterogeneities of IgH genes of gastric MALT lymphoma before and after the eradication of *H. pylori* may hold to a clue for further understanding of the characteristics of the development and progression of this kind of MALT lymphoma.

As for the recombination of Ig genes, the fact that the accumulation of somatic mutations shows a much higher replacement (R)/silent (S) ratios in the CDRs than the FRs in the V genes indicates that the B cell has undergone positive antigen selection during the germinal-centre reaction (Stewart and Schwartz, 1994). Assessment of the pattern of the IgH mutations responsible for *H. pylori* in MALT lymphoma determined that all of our cases of gastric MALT lymphoma displayed a hypermutation mechanism, as also previously reported (Qin *et al*, 1995; Du *et al*, 1996). Furthermore, the disappearance pf monoclonarity in the clones from cases 1 and 2 after the eradication of *H. pylori* indicated a tendency to display a mutation pattern by positive antigen selection, because the number of R mutations was higher in the CDRs than could be expected by chance, especially in case 1 (*P*<0.05). In addition to the frequency of hypermutation of IgH genes, the mutation pattern of positive selection may also reflect the development of MALT lymphoma status dependent on *H. pylori*. As for V gene usage, the lymphoma cells in four of the five cases used V3 genes, and the remaining one used a V4 family gene. With the exception of case 2, the percentages of homology to their closest germline counterparts are varied, so that there was no preferential usage of the V gene could be observed.

In cases 3–5, low intraclonal heterogeneity of the IgH genes in residual clones after the eradication of *H. pylori* was low, which seems to imply the expansion of an independent tumour clone on *H. pylori*. Although complete eradication of *H. pylori* was achieved without relapse, in case 1 with the biclonal tumour-derived clone, case 1b had no intraclonal sequence heterogeneity before treatment, so it is conceivable that an expansion had already occurred. In case 4, tumour cells were treated with cyclophosphamide before the second analysis of IgH gene, so that the treatment may have affected the low intraclonal heterogeneities. Case 5 had a high-grade component and karyotypic abnormality in the form of trisomy 3 (data not shown) and was considered to consist of the transformed DLBCL tumour cell clones. The equivalent of the remaining low intraclonal heterogeneities is still unclear and warrants further investigation.

In conclusion, low-grade gastric MALT lymphomas seem to expand *in vivo* due to an ongoing mutator of *H. pylori in vivo*. The characteristic feature of tumour clones obtained from residual tumours after the eradication of *H. pylori* seems to be a very low intraclonal heterogeneity, which may potentially be independent of *H. pylori*. This characteristic feature of these clones need to be considered as an important factor in the clinical management of patients with gastric MALT lymphomas.

## Figures and Tables

**Table 1:** Clinical, histological and laboratorical characteristics of gastric MALT lymphomas

				**Histological score**		**PCR analysis**		
**Case**	**Age (years)/sex**	**Endoscopic appearance**	**Phenotype**	**Before eradication of *H. pylori***	**After eradication of *H. pylori***	**Before eradication of *H. pylori***	**After eradication of *H. pylori***	**Months after treatment of *H. pylori***
1	78/F	Single ulcer with fold hypertrophy	IgM *λ*	5	2	Monoclonal	Polyclonal	17
2	65/F	Small erosion	IgM *κ*	4	2	Monoclonal	Polyclonal	10
3	63/F	Granular	ND	5	5	Monoclonal	Monoclonal	6
4	53/M	Multiple ulcer	ND	5	5	Monoclonal	Monoclonal	20
5^a^	67/M	Single ulcer	IgMD *λ*	4	5	ND	Monoclonal	30

MALT=mucosa-associated lymphoid tissue; ND=not determined.

a After eradication of *H. pylori*.

**Table 2:** Analysis of V_H_ genes from cases of MALT lymphoma before eradication

				**Mutations**						
**Case**	**GL donor**	**V_H_ family**	**% Homology**	**FR**	**R/S**	**CDR**	**R/S**	**CDR/FR**	**D segment**	**J_H_ segment**
1a	YAC-9	V_H_3	93.7	5	0.7	5	>5	1	D21/9-DAI or DA4	J_H_4
1b	DP-77	V_H_3	88.2	8	1.7	8	7	1.3	H23-D-DLR1	J_H_4
2	DP-77	V_H_3	98.2	1	>1	2	>2	2	H23-D reverse	J_H_4
3	DP-46	V_H_3	96.4	3	2	3	>3	1	DN1	J_H_4
4	DP-54	V_H_3	95.4	4	3	2	>2	0.5	DXP′l	J_H_4
5^a^	V4–31	V_H_4	92.0	6	2	2	>2	0.3	DHQ52	J_H_4

MALT=mucosa-associated lymphoid tissue; GL=germ line. When all mutations induced replacement (S=0), results are exposed as > number of replacements observed.

a After eradication of *H. pylori.*

**Table 3:** Distribution of mutations in the tumour-derived V_H_ sequences

		**Expected**		**Observed**		
**Case**	**Region**	**R**	**S**	**R**	**S**	***P* a**
1a	CDR	2	1	5	0	
						0.04
	FR	5	2	2	3	
						
1b	CDR	4	1	7	1	
						0.02
	FR	8	3	5	3	
						
2	CDR	0	0	2	0	
						0.10
	FR	2	1	1	0	
						
3	CDR	2	0	3	0	
						0.09
	FR	3	1	2	1	
						
4	CDR	2	0	2	0	
						0.27
	FR	3	1	3	1	
						
5	CDR	2	0	2	0	
						0.30
	FR	4	2	4	2	

a *P* is the probability of obtaining the number of R mutations found in the CDR by chance.

**Table 4:** Frequency of intraclonal sequence heterogeneity before and after treatment for *H. pylori*

	**Before treatment**		**After treatment**	
**Case**	**Tumour-derived sequences/clones sequenced**	**Frequency of sequence heterogeneity (%)**	**Tumour-derived sequences/clones sequenced**	**Frequency of sequence heterogeneity (%)**
1a	5/11	0.37	—	—
1b	5/11	0.00	—	—
2	2/11	0.25	—	—
3	8/9	0.30	4/6	0.12
4	6/6	0.49	6/6	0.10
5	ND	ND	10/10	0.18

ND=not determined.
